# Enhancing Adult Women’s Pelvic Health Knowledge Through Virtual Education: A Pilot Study

**DOI:** 10.1093/geroni/igaf122.2736

**Published:** 2025-12-31

**Authors:** LaVona Traywick, Alexis Gillett, Anna Dold, Kara LaGorio

**Affiliations:** University of Oklahama, Oklahoma City, Oklahoma, United States; Alice Walton School of Medicine, Bentonville, Arkansas, United States; Arkansas Colleges of Health Education, Fort Smith, Arkansas, United States; Arkansas Colleges of Health Education, Fort Smith, Arkansas, United States

## Abstract

Providing evidence-based care is essential to optimizing healthcare outcomes, particularly for senior adult women in underserved areas. Older women often face pelvic health challenges, including urinary tract infections (UTIs), pelvic floor dysfunction, pelvic organ prolapse, urinary incontinence, and chronic pelvic pain. Access to healthcare in rural areas remains a significant barrier, particularly for homebound seniors. Virtual education offers a promising avenue for addressing these disparities. This study evaluated the impact of a virtual pelvic health education session on knowledge retention among women in an underserved area. Forty-two female participants were recruited in Western Arkansas. Participants completed an online pre-session questionnaire (pre) to assess baseline pelvic health knowledge, attended a single virtual education session led by a healthcare provider specializing in pelvic health, and completed an on-line post-session (post-1) knowledge questionnaire, and one month post virtual (post-2) interview. The results demonstrate a significant increase in pelvic health knowledge immediately after the education session post-1 (p < 0.001) and in the follow-up post-2 (p < 0.001) assessment. Thematic interview results indicated that learning about pelvic health in a virtual environment was a positive experience. Thus, virtual education sessions appear to be a viable strategy for enhancing pelvic health knowledge, particularly among rural and homebound senior women with limited access to healthcare. This approach has the potential to empower older women to address pelvic health concerns proactively, thereby improving health outcomes and quality of life. Additionally, telehealth and virtual healthcare sessions provide significant benefits, including increased accessibility, convenience, cost-effectiveness, and the ability to reach underserved populations.

